# Concomitant incarcerated right direct inguinal hernia and right femoral hernia in a male patient: a case report

**DOI:** 10.1093/jscr/rjaf391

**Published:** 2025-06-27

**Authors:** Aseel Shams, Hanin Attar, Abdulqader Almuallim, Faris Alsobyani, Ahmed Gadah, Samah Khayyat

**Affiliations:** General Surgery Department, Alnoor Specialist Hospital, 3rd Ring Road, Alhijrah, Makkah 24241, Saudi Arabia; General Surgery Department, Alnoor Specialist Hospital, 3rd Ring Road, Alhijrah, Makkah 24241, Saudi Arabia; General Surgery Department, Alnoor Specialist Hospital, 3rd Ring Road, Alhijrah, Makkah 24241, Saudi Arabia; General Surgery Department, Alnoor Specialist Hospital, 3rd Ring Road, Alhijrah, Makkah 24241, Saudi Arabia; General Surgery Department, Alnoor Specialist Hospital, 3rd Ring Road, Alhijrah, Makkah 24241, Saudi Arabia; General Surgery Department, Alnoor Specialist Hospital, 3rd Ring Road, Alhijrah, Makkah 24241, Saudi Arabia

**Keywords:** inguinal hernia, femoral hernia, mesh repair, Richter, Amyand’s, concomitant, incarcerated

## Abstract

Concomitant indirect inguinal and femoral hernias on the same side are rare and often undetected preoperatively, especially when complicated by incarceration. We report this case to highlight its rarity and importance in the clinical decision process. An 83-year-old male with asthma presented with right inguinal pain and vomiting. The workup revealed a right direct inguinal hernia with small bowel obstruction. During surgery, a right direct inguinal hernia with a non-inflamed appendix (Amyand's hernia) and a right femoral hernia with a small bowel segment (Richter's hernia) were found. Both hernias were repaired with mesh. Concomitant right indirect inguinal and femoral hernias are challenging to diagnose preoperatively, especially in males. Early surgical intervention, aided by high suspicion and anatomical knowledge, prevents complications. This case demonstrates successful treatment with open mesh repair of both hernias.

## Introduction

Concomitant indirect inguinal and femoral hernias on the same side is not common [1]. A preoperative examination may not be able to detect the coexisting conditions, especially in the setting of complications such as the incarceration of the hernia. Inguinal hernia repair is an extremely common operation performed by surgeons. Inguinal hernias account for 75% of all abdominal wall hernias. Two-thirds of these hernias are indirect, making an indirect hernia the most common groin hernia in both males and females. Males account for about 90% of all inguinal hernias. Femoral hernias account for only 3% of all inguinal hernias. On the other hand, the majority of femoral hernias are four times higher in women than in men [2]. This discrepancy in frequency can lead to unexpected intraoperative results that can be predicted and planned in anticipation, which adds value to our case report in the literature as a whole to emphasize this finding.

## Case presentation

An 83-year-old man with a history of asthma presented to the emergency department complaining of right inguinal pain. A language barrier prevented a detailed history. The pain had recurred for 2 years and became more severe 3 days before admission. He experienced vomiting of food contents and poor oral intake. He denied fever, anorexia, or urinary symptoms.

On examination, his abdomen was soft with mild tenderness in the right lower quadrant. A non-reducible right groin swelling with a negative cough impulse was noted, with no overlying skin changes. A digital rectal examination revealed an empty rectum. There was a scar on the left side from a previous open inguinal hernia repair with mesh. He reported no other surgical history or chronic conditions apart from asthma.

**Figure 1:**
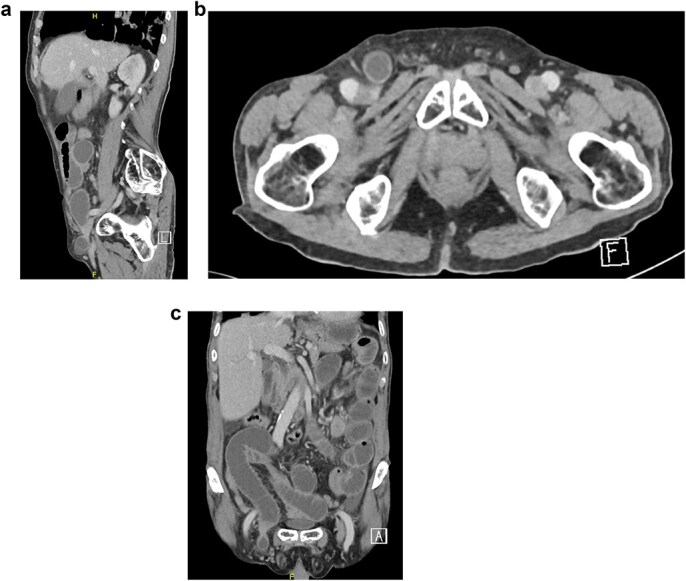
(a–c) Direct inguinal hernia.

Bloodwork was normal except for a leukocyte count of 15 × 10^9^/l. A chest x-ray was unremarkable. An abdominal X-ray showed dilated small bowel loops with multiple air-fluid levels. Abdominal computed tomography (CT) with intravenous (IV) contrast confirmed a right-sided direct inguinal hernia with small bowel obstruction, preserved bowel enhancement, and no extraluminal air. However, a femoral hernia was not identified on imaging before surgery or on retrospective review postoperatively (Fig. 1a–c). The patient received 2 g of IV cefazolin preoperatively.

**Figure 2:**
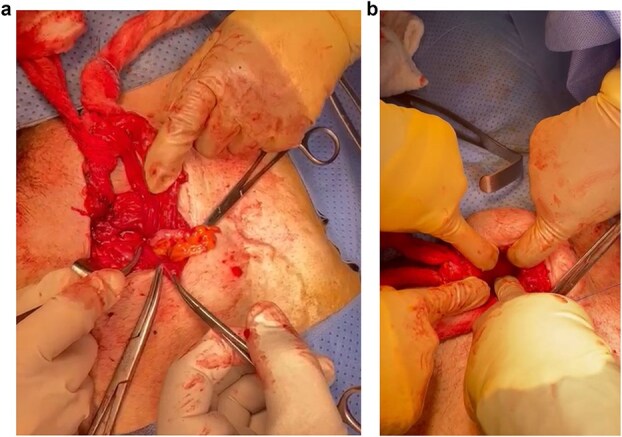
(a) Amyand’s direct inguinal hernia containing a non-inflamed appendix. (b) Femoral hernia.

During surgery, a right direct inguinal hernia sac contained a non-inflamed appendix (Amyand’s). Another bulge beneath the inguinal ligament was identified as a right femoral hernia containing the antimesenteric portion of the small bowel (Richter’s). An open appendectomy was done, then an open McVay hernia repair with polypropylene mesh was performed to cover both defects (Fig. 2a and b) via a single 11 × 6 cm mesh, which was fixed in place via Prolene sutures. The repair includes opening of the transversalis fascia. Coverage of inguinal and femoral orifices was ensured. The patient was discharged on postoperative day 3 in stable condition but was lost to follow-up after returning to his home country.

## Discussion

A hernia is a protrusion of the abdominal wall that may contain part of the viscus [1]. Inguinal hernias have various classification systems, yet there is no single universally accepted one. Among the commonly utilized methods is the Nyhus classification, which sorts hernia defects based on their size, location, and type.

The Nyhus Classification System is outlined as follows:

Type I:

- An indirect hernia with a normal-sized internal ring, typically found in infants, children, and small adults.

Type II:

- An indirect hernia with an enlarged internal ring but without pressing on the floor of the inguinal canal, and it does not extend into the scrotum.

Type III-A:

- A direct hernia where the size is not considered.

Type III-B:

- An indirect hernia that has grown sufficiently to infringe upon the posterior inguinal wall. Indirect sliding or scrotal hernias are often placed in this category due to their association with an extension into the direct space. This type also encompasses pantaloon hernias.

TYPE III-C:

- A femoral hernia.

Type IV:

- A recurring hernia; sometimes modifiers A to D are added, corresponding to direct, indirect, femoral, or mixed, respectively [3].

Generally, inguinal and groin hernias present as a, which may cause a dragging sensation. Painful lumps are usually due to incarceration and obstruction or strangulation and will present with colicky abdominal pain and vomiting. Groin hernias are common disorders with various variations and may be difficult to diagnose preoperatively. As a result, the nature and number of hernias observed in surgery may differ from those identified at diagnosis. The femoral hernia was reducible in our case, which might explain why it was not visible on CT, leading to further uncertainty preoperatively. Femoral hernia is more susceptible to strangulation than inguinal hernias, and there are identifiable risk factors associated with groin hernias. Some identifiable risk factors can increase the occurrence of groin hernias and are divided into two general categories, namely patient-related risk factors and external risk factors [4]. These factors include smoking, increased intraabdominal pressure, systemic connective tissue disorders, low body mass index, old age, and male gender [4]. In this case, the patient was a male with advanced age and asthma, and these were the underlying risk factors. Different imaging modalities are used to confirm hernia diagnosis, including ultrasonography, CT, and magnetic resonance imaging (MRI) [3].

In this case, CT only showed us a right-sided direct inguinal hernia with small bowel obstruction. Operative management is the mainstay of treatment to repair the defects as soon as possible. This can either be done laparoscopically or through open approaches. Open approaches include McVay's (high), Lotheissen's (trans-inguinal), and Lockwood's (low), among others [5]. In this case, the open technique was used to repair the hernias. An open inguinal approach was done, and there was an intraoperative finding of mesh from an old repair and Amyand’s hernia. After the delivery of the sac, there was an incidental finding of a concomitant femoral Richter’s hernia. Both defects were fixed with the application of a mesh.

## Conclusion

Concomitant right indirect inguinal hernia and right femoral hernia are rare conditions that can be challenging to diagnose preoperatively, particularly in male patients [4]. However, a high index of Suspicion with good anatomical knowledge will help in early surgical intervention, which is essential to prevent complications such as incarceration and strangulation. In this case report, the patient was successfully treated with open mesh repair of both hernias.

## Data Availability

All data mentioned within this manuscript can be provided as requested.
